# Dismal prognosis of *Pneumocystis jirovecii* pneumonia in patients with multiple myeloma

**DOI:** 10.1007/s00277-023-05586-8

**Published:** 2023-12-20

**Authors:** C Riedhammer, J Düll, C Kestler, S Kadel, J Franz, P Weis, F Eisele, X Zhou, M Steinhardt, L Scheller, J Mersi, J. M Waldschmidt, H Einsele, D Turnwald, K. M Kortüm, G Surat, L Rasche

**Affiliations:** 1 Department of Internal Medicine II, University Hospital of Würzburg, Würzburg, Germany; 2 Institute for Diagnostic and Interventional Radiology, University Hospital of Würzburg, Würzburg, Germany; 3 Institute of Hygiene and Microbiology, University of Würzburg, 97080 Würzburg, Germany; 4 Unit for Infection Control and Antimicrobial Stewardship, University Hospital of Würzburg, 97080 Würzburg, Germany

**Keywords:** *Pneumocystis jirovecii* pneumonia, Multiple myeloma, PJP prophylaxis

## Abstract

Patients with multiple myeloma (MM) are at high risk for infections, including opportunistic infections such as *Pneumocystis jirovecii* pneumonia (PJP). We conducted a retrospective analysis of patients with MM developing PJP over a 6-year period between January 2016 and December 2021 at the University Hospital of Würzburg by screening cases of microbiologically documented PJP. A total of 201 positive results for *P. jirovecii* in respiratory specimens were retrospectively retrieved through our microbiology database. Of these cases, 13 patients with MM fulfilled the definition of probable PJP according to EORTC fungal disease definitions. We observed two peaks in PJP incidence, one after stem cell transplantation during first-line treatment (*n* = 5) and the other in heavily pretreated patients with six or more prior lines of therapy (*n* = 6). There was high morbidity with nine (69%) patients admitted to the ICU, seven of whom (78%) required mechanical ventilation, and high mortality (62%, *n* = 8). Notably, only two of the 13 patients (15%) had received PJP prophylaxis. The main reason for discontinuation of prophylaxis with trimethoprim-sulfamethoxazole was grade IV neutropenia. The observed morbidity and mortality of PJP in MM patients are significant and even higher than reported for patients with other hematologic malignancies. According to most current guidelines, the use of prophylaxis would have been clearly recommended in no more than three (23%) of the 13 patients. This illustrates the need to critically reconsider the indications for PJP prophylaxis, which remain incompletely defined.

## Introduction

Immune dysfunction and related infectious complications are a hallmark of multiple myeloma (MM), the second most common hematologic malignancy. Both cellular and humoral immune responses are compromised by the disease itself [1] but also by its treatment, leaving MM patients susceptible to all types of infections, including those caused by opportunistic pathogens [2].

A serious and often fatal opportunistic infection in immunocompromised hosts is *Pneumocystis jirovecii* pneumonia (PJP). Currently, the incidence of PJP is decreasing in HIV-positive patients, but increasing in HIV-negative patients, who experience a more aggressive disease course with a higher need for intensive care treatment and a higher proportion of fatal outcomes [3–5]. Diagnosis of PJP is based on host factors and typical clinical, radiological, and microbial findings. According to the EORTC criteria, the diagnosis of *proven PJP* requires the presence of clinical and radiological criteria and microscopic demonstration of *P. jirovecii* in respiratory specimens or tissue [6]. *Probable PJP* is based on the presence of appropriate host factors, clinical and radiological criteria, amplification of *P. jirovecii* DNA in respiratory specimens, and/or detection of β-D-glucan in serum [6].

Trimethoprim/sulfamethoxazole (TMP-SMX) is the treatment of choice for PJP, alternative options include atovaquone, pentamidine, and clindamycin-primaquine [3, 7]. For the prevention of PJ disease, current ASCO, ECIL, and national guidelines explicitly recommend PJP prophylaxis only in hematologic patients at high risk, such as those following allogeneic transplantation, those diagnosed with acute lymphoblastic leukemia, or those receiving high-dose corticosteroids or certain lymphodepleting agents [7–10]. However, some MM-specific guidelines also clearly recommend PJP prophylaxis in patients following autologous hematopoietic stem cell transplantation (HSCT) [11, 12], which is also the standard of care at our institution.

To our knowledge, PJP has yet to be analyzed systematically in patients with MM. This becomes even more important as novel treatment options such as CAR-T-cell therapies and bispecific antibodies put more patients at risk for opportunistic infections like PJP. In studies with BCMA-directed bispecific antibodies, a significant proportion (3.6–4.9%) of patients developed PJP [13]. Therefore, we retrospectively collected all cases of PJP in myeloma patients from 2016 to 2021 at our institution, a tertiary care hospital, and evaluated patient characteristics, diagnostic findings, disease course and outcome, and the use of PJP prophylaxis as a quality improvement project in collaboration between the in-house antimicrobial stewardship team (AMS) and the hematology department.

## Methods

All cases of microbiologically documented PJP (positive immune staining and/or positive RT-PCR) in respiratory specimens were retrospectively collected between January 2016 and December 2021 through our microbiology database and screened for patients with a diagnosis of MM. The clinical history of MM patients was then retrieved.

Diagnoses of *probable* and *proven* PJP were assessed according to EORTC/MSGERC invasive fungal disease definitions in individuals without HIV [6]. As β-D-glucan testing was not available during the entire study period, it was not considered in the diagnosis of additional cases with probable PJP. Quantitative PCR thresholds for distinguishing between colonization and infection with *P. jirovecii* have not yet been established, so PCR thresholds were not used in this analysis.

## Results

### Patient characteristics

To identify patients with MM and PJP, we reviewed 201 cases with positive tests for *P. jirovecii* in respiratory specimens through our microbiology database. Nine of these 201 cases showed a positive microscopy for *P. jirovecii*; the remaining 192 cases had been tested positive by qPCR. Thirteen patients with a diagnosis of MM, all with positive qPCR and typical clinical and radiological criteria, met the criteria for *probable PJP* according to the EORTC guidelines [6]. The median age at diagnosis of PJP was 60 years. Thirty-eight percent of patients were male.

We observed two peaks of PJP incidence, one after HSCT during first-line treatment (*n* = 5, 38%) and the other in heavily pretreated patients with six or more prior lines of therapy (*n* = 6, 46%). Patient characteristics, diagnostic findings, treatment, and outcome are shown in Table 1. Patients had a median history of multiple myeloma diagnosis of 5.8 years. Six (46%) patients had received corticosteroids in the 4 weeks preceding the diagnosis of PJP with a median dosage of 13 mg prednisolone/day. A total of four patients had undergone prior allogeneic transplantation, two as part of a primary tandem autologous-allogeneic transplantation strategy and two as a salvage therapy for refractory disease.

**Table 1:** Patient characteristics, key diagnostic findings, outcome, and use of PJP prophylaxis

MM patients not surviving PJP																	
ID	Gender	Age	MM history in years	Last or current MM treatment	LOT	Mean PSL dose (mg)/days last 4 weeks	LDH [U/l]	CT value *P. jirovecii* in qPCR	CD4^+^ T cells	CT scan	Viral coinfections	Use of PPX	Retrosp. PPX-indication (ECIL/ASCO)	Anti-microbial PJP treatment	ICU	IMV	Outcome
MM-9	F	66	0.7	HDM/ASCT 3 weeks before	1	0	395	28.4	43/ μL	GGO + CON	No	No (discontinued due to grade IV neutropenia)	No	IV TMP-SMX 21 days + PSL	Yes	Yes	Deceased (d35)
MM-1	F	61	0.8	Allo-SCT 2 months before	1	0	305	28.6	N/A	GGO	HSV	Atovaquone 750 mg bid	Yes	IV TMP-SMX 4 days, then atovaquone 750 mg bid + PSL	Yes	Yes	Deceased (d15)
MM-7	F	65	2.2	DVd (start 2 weeks before)	2	33	351	31.2	N/A	GGO + CON	CMV + HSV	Pentamidine p.i. planned but not yet started	Yes	IV TMP-SMX 1 day + PSL	Yes	No	Deceased (d1)
MM-6	M	80	17.5	ERd	2	14	560	18.4	N/A	GGO	CMV	No	No	IV TMP-SMX 7 days + PSL	Yes	Yes	Deceased (d7)
MM-8	M	60	10.0	Pom-PAD 4 months before	8	0	1481	22.6	19/ μL	CON	No	TMP-SMX 960 mg 3×/week	No	None (diagnosis post mortem)	Yes	Yes	Deceased pre-diagnosis
MM-11	F	55	10.0	Allo-SCT 5 weeks before	8	0	618	30.6	N/A	GGO + CON	No	No (discontinued due to grade IV neutropenia)	Yes	IV TMP-SMX + PSL	Yes	Yes	Deceased (d4)
MM-13	F	79	11.0	Blenrep	8	7	600	N/A	131/ μL	GGO + CON	No	No	No	IV TMP-SMX 3 days	No (DNR/DNI order)	No	Deceased (d3)
MM-3	F	58	11.0	Blenrep-Vd 2 months before	11	0	426	20.2	N/A	GGO + CON	No	No	No	IV TMP-SMX 9 days + PSL	Yes	Yes	Deceased (d9)
Summary (median)		**63**	**10.0**		**5**		**493**				**3/8 (38%)**	**2/8 (25%)**	**3/8 (38%)**		**7/8 (88%)**	**6/8 (75%)**	
MM patients surviving PJP																	
ID	**Gen-der**	**Age**	**MM history in years**	**Last or current MM treatment**	**LOT**	**Mean PSL dose (mg)/days last 4 weeks**	**LDH [U/l]**	**CT value** ***P. jirovecii*** **in qPCR**	**CD4**^**+**^ **T cells**	**CT scan**	**Viral coinfections**	**Use of PPX**	**Retrosp. PPX-indication (ECIL/ASCO)**	**Anti-microbial PJP treatment**	**ICU**	**IMV**	**Outcome**
MM-4	M	70	0.7	HDM/ ASCT 3 weeks before	1	0	304	20.9	N/A	GGO	No	No (not started for fear of prolonging leukopenia during HDM)	No	Atovaquone 9 days, then IV TMP-SMX 6 days	Yes	Yes	Survived
MM-12	M	59	2.1	EKRd (clinical trial)	1	5	290	24.1	N/A	GGO + CON	No	No	No	IV TMP-SMX 4d, PO TMP-SMX 17 days	No	No	Survived
MM-2	F	55	4.5	Allo-SCT 2 years before	1	0	207	23.8	N/A	GGO + CON	No	No (stopped 3 months before, CD4^+^ T cells ≥ 200/ μL)	No	IV TMP-SMX 12 days	No	No	Survived
MM-5	M	59	5.8	Pom-PAD	6	13	285	32.8	26/µL	GGO + CON	No	No	No	Clindamycin/Caspofungin + PSL	No	No	Survived
MM-10	F	57	8.0	DRd	6	13	268	28.0	N/A	GGO + CON	No	No	No	IV TMP-SMX 30 days + PSL	Yes	No	Survived
Summary (median)		**59**	**4.5**		**1**		**285**				**0/5 (0%)**	**0/5 (0%)**	**0/5 (0%)**		**2/5 (40%)**	**1/5 (20%)**	

*Allo-SCT* allogeneic hematopoietic stem cell transplantation, *blenrep* belantamab mafodotin, *CON* consolidations, *DRd* daratumumab, lenalidomide, and dexamethasone, *E(K)Rd* elotuzumab plus (carfilzomib/)lenalidomide/dexamethasone, *GGO* ground glass opacities, *HDM/ASCT* high-dose melphalan followed by autologous transplantation, *ICU* intensive care unit, *IMV* invasive mechanical ventilation, *LOT* lines of therapy, *Pom-PAD* pomalidomide, bortezomib, doxorubicin, dexamethasone, *PPX* prophylaxis, *PSL* prednisolone, *Vd* bortezomib and dexamethasone

### Diagnostic findings, disease course, and outcome

PJP is a diagnostic challenge. All but one patient had elevated LDH with a median of 351 U/l. Four patients had a known CD4+ T cell count, all below 200 /μL with a range of 19 to 131 /μL. Chest CT scans of all patients showed typical radiological findings such as ground glass opacities (GGO) in all but one patient (92%) and/or additional consolidations in 10 patients (77%) as a sign of a higher grade of inflammation. qPCR Ct values ranged from 18.4 to 32.

Antimicrobial treatment for *P. jirovecii* was given to 12 out of 13 patients. The patient who was not treated for PJP deteriorated rapidly and was diagnosed with PJP post mortem. Ten out of 13 patients (77%) were treated primarily with high-dose TMP-SMX according to the standard of care [3]. One patient, in whom PJP was diagnosed during grade IV neutropenia following high-dose chemotherapy, was first treated with atovaquone and switched to TMP-SMX after stable engraftment. One other patient was treated with clindamycin/caspofungin. Adjunctive steroids were used in 8 out of 13 patients (62%).

Nine of the 13 patients (69%) were admitted to the ICU, two of them underwent non-invasive ventilation with high-flow oxygen and seven of nine patients (78%) required mechanical ventilation. Duration of mechanical ventilation ranged between 1 and 10 days. Eight of the 13 patients (62%) died, seven of them due to respiratory failure. The other patient showed altered mental status and seizures of unresolved etiology. The patients with a fatal outcome had a longer history of MM, were more heavily pretreated, and showed higher baseline LDH levels (Table 1). Three of them also had parallel reactivation of HSV and/or CMV (Table 1). Only one of the patients surviving PJP required ICU treatment. Together, our results highlight the severe nature of PJP in MM.

### Use of prophylaxis

A key question is whether prophylaxis was used in the MM patients who acquired PJP. Remarkably, only two (13%) patients were on a PJP prophylaxis, one on TMP-SMX and the other on atovaquone. According to most national and international guidelines [8–10], prophylaxis would have been strictly indicated in only three (23%) of the 13 patients. One of them was the patient receiving atovaquone, the second patient developed PJP before the planned pentamidine prophylaxis could be started, and in the third patient, TMP-SMX had been interrupted for fear of prolonging chemotherapy-induced neutropenia. TMP-SMX had also been discontinued in two other patients with grade IV neutropenia following high-dose chemotherapy at least 3 weeks prior to diagnosis of PJP. In summary, neutropenia turned out to be an important factor in clinical decision-making regarding the discontinuation of prophylaxis in our study.

## Discussion

This retrospective study analyzes *Pneumocystis jirovecii* pneumonia in patients with MM in a single center over the course of 6 years. To date, there are very few case reports of up to three patients dedicated to PJP in MM [2, 14–16]. In our patient population, PJP mainly affected MM patients on their first line of therapy after HSCT and heavily pretreated patients with six or more prior lines of therapy. One possible explanation might be that a low CD4+ cell count is a risk factor for PJP even in HIV-negative patients [17] and that CD4+ cell counts decrease with each line of myeloma therapy [2]. The observed two incidence peaks in early and late disease stages are therefore likely to represent the time points when physicians treating MM patients ought to be particularly aware of the occurrence of opportunistic infections like PJP.

We observed very poor outcomes in MM patients with a high ICU admission rate of 69% of patients, 78% of them requiring mechanical ventilation. Other studies have shown lower ICU admission rates of 42% for patients with hematologic malignancy [18]. Reported ICU admission rates for HIV-negative patients range from 38 to 52%, with 71% of them requiring mechanical ventilation [19, 20]. A rate of 43% of allogeneic transplant recipients who develop PJP has been reported to require mechanical ventilation [4]. Around two-thirds of our MM patients died within 4 weeks after PJP diagnosis or due to PJP-related complications. In the literature, PJP-related deaths have been reported in 43–48% of patients undergoing HSCT (autologous and allogeneic) [4, 21], in 29% of patients with hematologic malignancy in general [18], and in 15–30% of HIV-negative patients [4, 22, 23]. In summary, morbidity and mortality in MM patients were higher than reported for hematologic patients and HIV-negative patients.

The key question arising from this is what prophylactic measures are adequate to prevent the occurrence of potentially fatal PJP in MM patients. The use of TMP-SMX prophylaxis has been generally shown to be effective in reducing the risk of PJP by 85% [24]. Most MM patients in this analysis (77%, *n* = 10) did not have an indication for PJP prophylaxis according to most current national and international guidelines [8–10], recommending primary PJP prophylaxis in adult patients with high risk for PJP only. In all other settings, the use of PJP prophylaxis remains an individual decision, also due to a lack of prospective data in HIV-negative patients. Contrary to the aforementioned guidelines, the use of PJP prophylaxis in MM patients undergoing HSCT is recommended by MM-specific guidelines [11, 12] and practiced at many institutions, including ours. Our study also exemplifies fatal outcomes in which PJP prophylaxis was interrupted due to grade IV neutropenia. There are insufficient data regarding a possible prolongation of neutropenia with the prophylactic use of TMP-SMX; in a Cochrane analysis comparing studies on the use of PJP prophylaxis, there was no significant difference in adverse events between patients receiving TMP-SMX or placebo [24].

To conclude, PJP is a rare but potentially fatal complication in MM. Indications for PJP prophylaxis are still incompletely defined, resulting in inconsistent approaches. Discontinuation of PJP prophylaxis in neutropenic patients appears to be outcome-determining and needs to be critically reconsidered. Given the high morbidity and mortality of PJP in patients with MM in our analysis, indication for PJP prophylaxis should be re-evaluated at least in this subcohort, going along with new emerging treatments such as CAR-T-cells and bispecific antibodies. More randomized controlled trials are required to pave a different approach to prevent PJP.

## Data Availability

The datasets generated during and/or analysed during the current study are available from the corresponding author on reasonable request.

## References

[1] Pratt G, Goodyear O, Moss P (2007). Immunodeficiency and immunotherapy in multiple myeloma. Br J Haematol.

[2] Schütt P, Brandhorst D, Stellberg W, Poser M, Ebeling P, Müller S (2006). Immune parameters in multiple myeloma patients: influence of treatment and correlation with opportunistic infections. Leuk Lymphoma.

[3] Ibrahim A, Chattaraj A, Iqbal Q, Anjum A, Rehman MEU, Aijaz Z (2023). Pneumonia: a review of management in human immunodeficiency virus (HIV) and non-HIV immunocompromised patients. Avicenna J Med.

[4] Roux A, Canet E, Valade S, Gangneux-Robert F, Hamane S, Lafabrie A (2014). Pneumocystis jirovecii pneumonia in patients with or without AIDS, France. Emerg Infect Dis.

[5] Nüesch R, Bellini C, Zimmerli W (1999). Pneumocystis carinii pneumonia in human immunodeficiency virus (HIV)-positive and HIV-negative immunocompromised patients. Clin Infect Dis.

[6] Lagrou K, Chen S, Masur H, Viscoli C, Decker CF, Pagano L (2021). Pneumocystis jirovecii disease: basis for the revised EORTC/MSGERC invasive fungal disease definitions in individuals without human immunodeficiency virus. Clin Infect Dis.

[7] Cooley L, Dendle C, Wolf J, Teh BW, Chen SC, Boutlis C (2014). Consensus guidelines for diagnosis, prophylaxis and management of Pneumocystis jirovecii pneumonia in patients with haematological and solid malignancies, 2014. Intern Med J.

[8] Classen AY, Henze L, von Lilienfeld-Toal M, Maschmeyer G, Sandherr M, Graeff LD (2021). Primary prophylaxis of bacterial infections and Pneumocystis jirovecii pneumonia in patients with hematologic malignancies and solid tumors: 2020 updated guidelines of the Infectious Diseases Working Party of the German Society of Hematology and Medical Oncology (AGIHO/DGHO). Ann Hematol.

[9] Maertens J, Cesaro S, Maschmeyer G, Einsele H, Donnelly JP, Alanio A (2016). ECIL guidelines for preventing Pneumocystis jirovecii pneumonia in patients with haematological malignancies and stem cell transplant recipients. J Antimicrob Chemother.

[10] Taplitz RA, Kennedy EB, Bow EJ, Crews J, Gleason C, Hawley DK (2018). Antimicrobial prophylaxis for adult patients with cancer-related immunosuppression: ASCO and IDSA clinical practice guideline update. J Clin Oncol.

[11] Ludwig H, Kumar S (2023). Prevention of infections including vaccination strategies in multiple myeloma. Am J Hematol.

[12] Engelhardt M, Mertelsmann R, Duyster J, Engelhardt M, Mertelsmann R, Duyster J (2023). Konditionierung ASZT.

[13] Raje N, Anderson K, Einsele H, Efebera Y, Gay F, Hammond SP (2023). Monitoring, prophylaxis, and treatment of infections in patients with MM receiving bispecific antibody therapy: consensus recommendations from an expert panel. Blood Cancer J.

[14] Park H, Youk J, Kim HR, Koh Y, Kwon JH, Yoon S-S (2017). Infectious complications in multiple myeloma receiving autologous stem cell transplantation in the past 10 years. Int J Hematol.

[15] Dunphy L, Singh N, Keating E (2017) Multiple myeloma presenting with bilateral ankle pain (microangiopathy) and complicated by streptococcal meningitis and Pneumocystis carinii pneumonia. BMJ Case Rep. 10.1136/bcr-2016-217289 10.1136/bcr-2016-217289 PMC5307268 28174184

[16] Swan CD, Reid AB (2014). Three cases of presumed pneumocystis pneumonia in patients receiving bortezomib therapy for multiple myeloma. IDCases.

[17] Messiaen PE, Cuyx S, Dejagere T, van der Hilst JC (2017) The role of CD4 cell count as discriminatory measure to guide chemoprophylaxis against Pneumocystis jirovecii pneumonia in human immunodeficiency virus-negative immunocompromised patients: a systematic review. Transpl Infect Dis 19. 10.1111/tid.12651 10.1111/tid.12651 28035717

[18] Pagano L, Fianchi L, Mele L, Girmenia C, Offidani M, Ricci P (2002). Pneumocystis carinii pneumonia in patients with malignant haematological diseases: 10 years’ experience of infection in GIMEMA centres. Br J Haematol.

[19] Fillatre P, Decaux O, Jouneau S, Revest M, Gacouin A, Robert-Gangneux F (2014). Incidence of Pneumocystis jirovecii pneumonia among groups at risk in HIV-negative patients. Am J Med.

[20] Dunbar A, Schauwvlieghe A, Algoe S, van Hellemond JJ, Reynders M, Vandecasteele S (2020). Epidemiology of pneumonia and (non-)use of prophylaxis. Front Cell Infect Microbiol.

[21] Torres HA, Chemaly RF, Storey R, Aguilera EA, Nogueras GM, Safdar A (2006). Influence of type of cancer and hematopoietic stem cell transplantation on clinical presentation of Pneumocystis jirovecii pneumonia in cancer patients. Eur J Clin Microbiol Infect Dis.

[22] Bozzi G, Saltini P, Matera M, Morena V, Castelli V, Peri AM (2022). Pneumocystis jirovecii pneumonia in HIV-negative patients, a frequently overlooked problem. A case series from a large Italian center. Int J Infect Dis.

[23] Overgaard UM, Helweg-Larsen J (2007). Pneumocystis jirovecii pneumonia (PCP) in HIV-1-negative patients: a retrospective study 2002-2004. Scand J Infect Dis.

[24] Stern A, Green H, Paul M, Vidal L, Leibovici L (2014). Prophylaxis for Pneumocystis pneumonia (PCP) in non-HIV immunocompromised patients. Cochrane Database Syst Rev.

